# The Role of Personalization in Virtual Reality Exposure Therapy During the Treatment of Alcohol Use Disorder

**DOI:** 10.1192/j.eurpsy.2024.403

**Published:** 2024-08-27

**Authors:** V. J. J. A. Buwalda, B. Jansen, M. van Gisbergen, S. R. Rashnoodi

**Affiliations:** ^1^Psychiatry, GGD, Amsterdam; ^2^Psychiatry, Novadic Kentron, Vught; ^3^Academy of AI, Games, and Media, BUas, Breda, Netherlands

## Abstract

**Introduction:**

In Cue-Exposure-Therapy (CET), clients are exposed to triggers through objects, people and environments that arouse craving (Sinha et al. Neuropsychopharmacol. 2009;34 1198–1208). Virtual Reality Exposure therapy (VRET) is used to experience these triggers in a realistic, safe, and personalized way. VR has been used successfully in the treatment of psychiatric disorders. It has not yet been developed and sufficiently tested as an adjuvant in the clinical post-detoxification phase of treatment of alcohol use disorders (AUD) (e.g. Bordnick et al. Addict.Behav 2008;33 743-756; Hone-Blanchet et al. Front.Hum.Neurosci. 2014; 8(844) 1-15). Additionally, these treatment methods have been tested for effect, but not for effectiveness around different VR technologies (Ghita & Gutierrez-Maldonado. Addict.Behav 2018; 81 1-11; ). This study focuses on VRET-Recovry to examine to what extent VR worlds could be personalized in an effective manner to help treat AUD as well as clarifying on the ways in which the VR worlds could be optimized to achieve its goal.

**Objectives:**

The primary objectives of this study are to assess the necessity of personalization in VR environments for AUD treatment, identify the critical elements for personalization, and examine their impact on craving in AUD patients.

**Methods:**

The study included 10 AUD patients diagnosed according to DSM-V criteria, aged between 18 and 65, who were in the final week of clinical detoxification at a large addiction clinic in The Netherlands. A controlled experiment was conducted using the Recovry 1.0 VR system on Samsung Gear VR and Samsung Galaxy S9. The experiment involved exposure to various VR scenes (CG and 360o), including a neutral setting, a bar scene, and a home situation, with the duration and sequence controlled by a therapist. Data collection consisted of pre- and post-exposure questionnaires, heart rate and blood pressure measurements, and interviews.

**Results:**

Craving was remarkably low in the VR bar scene, primarily due to its unsociable context, limited alcohol visibility, and absence of peer pressure. Technical limitations, such as suboptimal resolution, also affected the feeling of presence. Positive results were shown that craving was predominantly stimulated in the apartment scene, driven by the presence of alcohol-related visual cues and social elements, resembling relaxed drinking with others.

**Conclusions:**

This study underscores that some degree of personalization is needed on all craving dimensions with clear preference was given to CG or 360°. The environments were dependent on the personal history and associations they represent to different levels of alcohol visibility (messy or clean), and types of drink (based on past drinking behavior), and different emotional contexts are needed (positive and negative).

**Disclosure of Interest:**

None Declared

